# Muscle cell identity requires Pax7-mediated lineage-specific DNA demethylation

**DOI:** 10.1186/s12915-016-0250-9

**Published:** 2016-04-13

**Authors:** Elvira Carrió, Alessandro Magli, Mar Muñoz, Miguel A. Peinado, Rita Perlingeiro, Mònica Suelves

**Affiliations:** Institut de Medicina Predictiva i Personalizada del Càncer (IMPPC) and Institut Germans Trias i Pujol (IGTP), Campus Can Ruti, 08916 Badalona, Spain; Lillehei Heart Institute, Department of Medicine, University of Minnesota, Minneapolis, 55455 USA

**Keywords:** DNA methylation, Cellular identity, Myogenesis, *Pax7*-induced ESCs, Apobec2, Epimarkers

## Abstract

**Background:**

Skeletal muscle stem cells enable the formation, growth, maintenance, and regeneration of skeletal muscle throughout life. The regeneration process is compromised in several pathological conditions, and muscle progenitors derived from pluripotent stem cells have been suggested as a potential therapeutic source for tissue replacement. DNA methylation is an important epigenetic mechanism in the setting and maintenance of cellular identity, but its role in stem cell determination towards the myogenic lineage is unknown. Here we addressed the DNA methylation dynamics of the major genes orchestrating the myogenic determination and differentiation programs in embryonic stem (ES) cells, their *Pax7*-induced myogenic derivatives, and muscle stem cells in proliferating and differentiating conditions.

**Results:**

Our data showed a common muscle-specific DNA demethylation signature required to acquire and maintain the muscle-cell identity. This specific-DNA demethylation is Pax7-mediated, and it is a prime event in muscle stem cells gene activation. Notably, downregulation of the demethylation-related enzyme Apobec2 in ES-derived myogenic precursors reduced myogenin-associated DNA demethylation and dramatically impaired the expression of differentiation markers and, ultimately, muscle differentiation.

**Conclusions:**

Our results underscore DNA demethylation as a key mechanism driving myogenesis and identify specific Pax7-mediated DNA demethylation signatures to acquire and maintain the muscle-cell identity. Additionally, we provide a panel of epigenetic markers for the efficient and safe generation of ES- and induced pluripotent stem cell (iPS)-derived myogenic progenitors for therapeutic applications.

**Electronic supplementary material:**

The online version of this article (doi:10.1186/s12915-016-0250-9) contains supplementary material, which is available to authorized users.

## Background

Developmental signals induce changes in gene expression and chromatin structure in order to define cell identity. Epigenetic mechanisms, and DNA methylation in particular, are essential for mammalian development being involved in the establishment and maintenance of cellular identity by creating cellular memory during embryonic development [1–3]. DNA methylation controls X-chromosome inactivation, expression of imprinted genes, represses the transcription of repeated sequences and prevents relocation of transposable elements (reviewed in [4]). In addition, DNA methylation affects gene expression by regulating promoters and distal regulatory elements, such as enhancers and insulators (reviewed in [5]). In the last few years, global analyses of DNA methylation dynamics in pluripotent stem cells and their differentiated progeny have begun to uncover the mechanisms involving coordinated changes occurring in the epigenome that are essential for lineage-specification and maintenance of cellular identity [6–15]. Very recently, we reported the genome-wide comparison of the DNA methylation profiles of pluripotent embryonic stem cells and muscle stem cells showing, in agreement with other studies, that cellular differentiation is associated with a modest but global increase in DNA methylation, which interestingly is accompanied by lineage-specific demethylation [16]. Although the mechanisms controlling DNA demethylation are still not well defined, several studies suggest the involvement of two major pathways in this process: the activity of TET methylcytosine dioxygenase proteins [17, 18] and AID/APOBEC cytidine deaminase enzymes [19–21]. Importantly, active DNA demethylation was reported in human muscle-specific genes in heterokaryons derived from the fusion of human non-muscle cells with mouse muscle cells, indicating a dynamic DNA methylation/demethylation interplay during muscle differentiation [22].

Skeletal muscle is the tissue accounting for the largest percentage of body mass and contributes to multiple body functions including the voluntary movements. The skeletal muscle is mainly composed of highly specialized and terminally differentiated multinuclear, post mitotic and contractile myofibres, and a small pool of muscle stem cells, known as satellite cells (SCs), which are essential for the growth, maintenance and regeneration of skeletal muscle throughout life (reviewed in [23]. The transcriptional regulatory network controlling the establishment of muscle embryonic progenitors in the myotome, as well as the activation of the satellite cells in the adult, involves upregulation of myogenic regulatory factors or MRFs (*Myf5*, *MyoD*, *Mrf4* and *Myog*), as well as silencing of other lineage-specifying genes (reviewed in [24]). In addition, members of the Pax gene family *Pax3* and *Pax7* lie upstream of MRFs and their expression is crucial to regulate muscle progenitor cell functions [25, 26]. *Pax7* is essential for the formation and maintenance of SCs, being expressed in quiescent and activated SCs, as well as in proliferating myogenic progenitors [27].

In the present study, we addressed for the first time the DNA methylation dynamics of the major genes orchestrating myogenic determination and differentiation by comparing pluripotent ESCs, myogenic precursors from *Pax7*-inducible ESCs, proliferating muscle stem cells, and their respective myotube derivatives. Our results showed a common muscle-specific DNA demethylation signature required to acquire and maintain the muscle-cell identity. Notably, downregulation of the muscle-specific cytidine deaminase Apobec2 in ES-derived myogenic precursors reduced myogenin-associated DNA demethylation and dramatically affected the expression of differentiation markers and, ultimately, muscle differentiation.

## Results

### DNA methylation profile of myogenic genes harbouring CpG island-promoters during myogenic differentiation

**Fig. 1:**
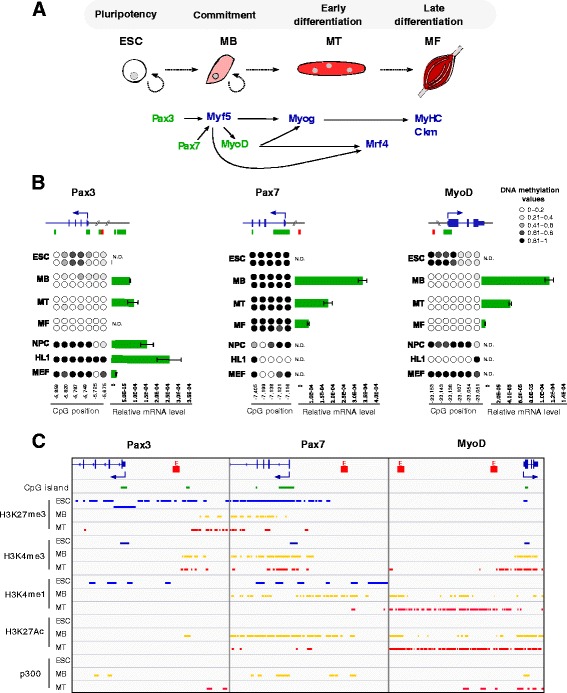
Epigenetic profile of myogenic genes harbouring a CpG island-promoter during myogenic differentiation. **a** Diagram of the myogenic differentiation model and the main genes driving myogenesis. CpG-rich and CpG-poor promoter genes are indicated in green and in blue, respectively. **b** Scheme of the *Pax3*, *Pax7* and *MyoD* genes and their associated CGIs (green) and enhancer regions (red). DNA methylation and expression of each gene were analysed in two biological duplicates of ESCs, myoblasts (MB), myotubes (MT), myofibres (MF), and NPC, HL1 and MEF cell lines. Each *circle* represents a CpG dinucleotide, and its position relative to the TSS is indicated below. The colour gradient represents the level of methylation indicated in the legend. *Bar charts* show gene expression values measured by qRT-PCR and normalized by *18S* expression (*n* = 2, mean ± SD). N.D. means non detectable. **c** Histone marks distribution and p300 binding in ESC (blue), MB (yellow) and MT (red) obtained from ENCODE Project and Dynlatch’s lab [38]. *CGIs* CpG islands, *ESCs* embryonic stem cells, *NPCs* neuronal precursor cells, *HL1* cardiomyocytes, *MEFs* mouse embryonic fibroblasts, *TSS* transcription start site

To elucidate DNA methylation dynamics associated with skeletal myogenic lineage commitment and terminal differentiation, we focused the analysis on genes known to be associated with this process: developmental genes (*Pax3* and *Pax7*), myogenic regulatory factors (*MyoD*, *Myog* and *Mrf4*) and terminal differentiation genes (*Myh1*, *Myh4*, *Myh8* and *Ckm*). Interestingly, these genes could also be classified according to their CpG distribution, which represent sites of potential DNA methylation. *Pax3*, *Pax7* and *MyoD* contain CpG islands (CGIs) in their promoters, belonging to the CpG-rich genes category; meanwhile, the other genes do not have CGI and their promoters are considered CpG-poor. Using bisulphite sequencing analysis, we compared the DNA methylation state of undifferentiated ESCs and muscle stem cells, isolated from adult skeletal muscle tissue, along with respective differentiated myotubes and mature myofibres (Fig. 1a). In addition, to assess whether the DNA methylation events were muscle-lineage specific, we analysed the methylation profiles of the following non-myogenic cell lines: neuronal precursor cells (NPCs), mouse embryonic fibroblasts (MEFs) and cardiomyocytes (HL1). As shown in Additional file 1a, b, all analysed CGIs in *Pax3* and *Pax7* regulatory regions were completely unmethylated in all the samples. Similar results were previously reported for MyoD CGI [28]. These results were expected since CGIs, usually located in the promoter regions of housekeeping and developmental genes, are known to be largely resistant to DNA methylation [29–31].

Since DNA methylation often occurs in non-CGI regions, we investigated whether enhancers and promoters present a cell-specific deposition of this modification. Previous studies of other groups identified two muscle-specific regulatory regions upstream of the MyoD transcription start site (TSS), located at -20 kb and -5 kb, respectively [32–34]. Importantly, it was shown that the distal *MyoD* enhancer located at -20 kb of the TSS was modulated by DNA methylation in mouse tissues [31]. Therefore, we analysed the methylation status of *Pax3* hypaxial somatic enhancer [35] located at -8 kb of the *Pax3* TSS, the *Pax7* region containing the critical RBP-Jκ binding site [36] located at -7,4 kb of *Pax7* TSS, and the two *MyoD* enhancer regions. As shown in Fig. 1b, although these regulatory regions were totally or partially methylated in ESCs and in non-muscle cells, *Pax3* and -20 kb *MyoD* enhancers were almost completely free of methylation in myogenic cells, which correlates with gene expression (Fig. 1b). On the contrary, the *Pax7* and -5 kb distal regulatory region of *MyoD* were both found highly methylated in muscle cells (Fig. 1b and Additional file 1c), suggesting that their activation would be independent of DNA methylation. Notably, NPCs and HL1 cells presented high levels of *Pax3* expression, despite high DNA methylation levels. This result would suggest that the *Pax3* hypaxial enhancer might be mainly associated to enhance *Pax3* expression in committed skeletal myogenic cells.

Next, to further characterize the epigenetic landscape involved in myogenic regulation, we took advantage of publicly available ChIP-seq data of histone post-translational modifications [37, 38]. As schematized in Fig. 1c, *Pax3*, *Pax7* and *MyoD* promoters showed a bivalent chromatin state characterized by histone 3 trimethylated on lysine 4 (H3K4me3) and lysine 27 (H3K27me3) in ESCs, which has been associated to poised transcription [39, 40]. This bivalent state was clearly resolved in favour of the positive mark H3K4me3 at myoblast (MB) and myotube (MT) stages for *MyoD*, whereas the developmental genes *Pax3* and *Pax7* retained the bivalent state (Fig. 1c). The analysis of these loci, including enhancer and distal regions, showed a gain in deposition of histone 3 monomethyl-lysine4 (H3K4me1), acetyl-lysine 27 (H3K27Ac) and increased recruitment of the p300 acetyltransferase at MB stage, and also at MTs in the case of *MyoD*. Since *MyoD* is already higher expressed in MBs, this maintenance of active enhancer marks in MTs might be involved in keeping an open chromatin state of the region to maintain gene expression, rather than promoting further transcriptional activation. All together, these results indicate that the unmethylated state of CGIs is conserved across the different cell types, whereas *Pax3* and -20 kb *MyoD* enhancer element show cell type-specific dynamic methylation patterns associated with changes in gene expression.

### Myogenic-specific promoters undergo DNA demethylation during muscle-lineage specification

**Fig. 2:**
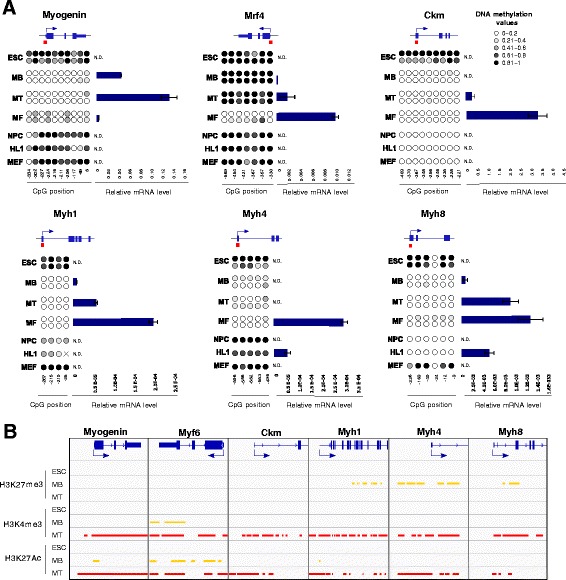
Epigenetic profile of muscle-specific CpG-poor promoter genes during myogenesis. **a** Scheme of the *Myog*, *Mrf4*, *Ckm* and *Myh1, 4* and *8* loci. Regions analysed by sodium bisulphite sequencing are indicated in red. Two biological replicates were performed for ESCs, myoblasts (MB), myotubes (MT) and myofibres (MF) and in NPC, HL1 and MEF cell lines. Each *circle* represents a CpG dinucleotide and its position relative to the TSS is indicated below. The colour gradient represents the level of methylation indicated in the legend. *Bar charts* show gene expression values measured by qRT-PCR and normalized by *18S* expression (*n* = 2, mean ± SD). N.D. means non detectable. **b** Histone marks distribution and p300 binding in ESC (blue), MB (yellow) and MT (red) obtained from ENCODE Project and Dynlatch’s lab [38]. *ESCs* embryonic stem cells, *NPCs* neuronal precursor cells, *HL1* cardiomyocytes, *MEFs* mouse embryonic fibroblasts, *TSS* transcription start site

Unlike *MyoD*, all the other MRFs *Myf5*, *Mrf4* and *Myog*, as well as the muscle differentiation markers display low CpG content promoters. The analysis of the promoter region, containing E-box and Mef2 binding sites, of *Myog* and the other differentiation genes *Ckm*, *Myh1*, *Myh4* and *Myh8* revealed high levels of methylation in ESCs and non-muscle cells, correlating with gene silencing, whereas the promoter regions in muscle cells were unmethylated (Fig. 2a). Interestingly, these differentiation genes became demethylated at the MB stage but remained silenced. These myogenic genes were not bivalent at the ESC stage and only presented active chromatin marks (H3K4me3 and H3K27Ac) in the promoter regions according to their expression timing. Strikingly, the terminal differentiation genes *Myh1*, *Myh4* and *Myh8* gained the repressive mark H3K27me3 in MB cells after DNA demethylation, suggesting that gene expression was maintained epigenetically repressed by PRC2 complex until the onset of the terminal differentiation stage (Fig. 2b). Interestingly, *Mrf4* followed a different DNA methylation pattern, being highly demethylated only in mature myofibres, where it is specifically highly expressed (Fig. 2a).

We also investigated the epigenetic profile of highly expressed pluripotency genes in ESC that become silenced in MB and MT. Genes containing a CGI overlapping their promoter region, such as Sox2 and Fgf4, were totally unmethylated in all cell types, whereas genes with CpG-poor content promoters, such as Dppa4 and Pou5f1, became highly hypermethylated upon cellular differentiation (Additional file 2a, b). Regarding histone marks, ESC showed high levels of H3K4me3 in the promoter regions of Sox2 and Fgf4, whereas MB and MT cells gained H3K27me3 in association with gene silencing (Additional file 2c). *Dppa4* and *Pou5f1* promoters presented also the active marks H3K4me3 and H3K27Ac. However, in differentiating cells the H3K27me3 mark was not acquired (*Dppa4*) or in a very small extent (*Pou5f1*), leaving the gain of DNA methylation as the postulated epigenetic silencing mechanism (Additional file 2c).

All together, these results suggest that myogenic differentiation genes become demethylated at CpG-poor regulatory regions during muscle-lineage determination, and are maintained silenced by the polycomb repressive histone mark H3K27me3 until gene activation. In contrast, pluripotency gene silencing involves complete DNA methylation or gain of H3K27me3 mark, depending on promoter CpG context.

### *Pax7*-induced ES-derived muscle differentiation recapitulates myogenic DNA methylation changes

**Fig. 3:**
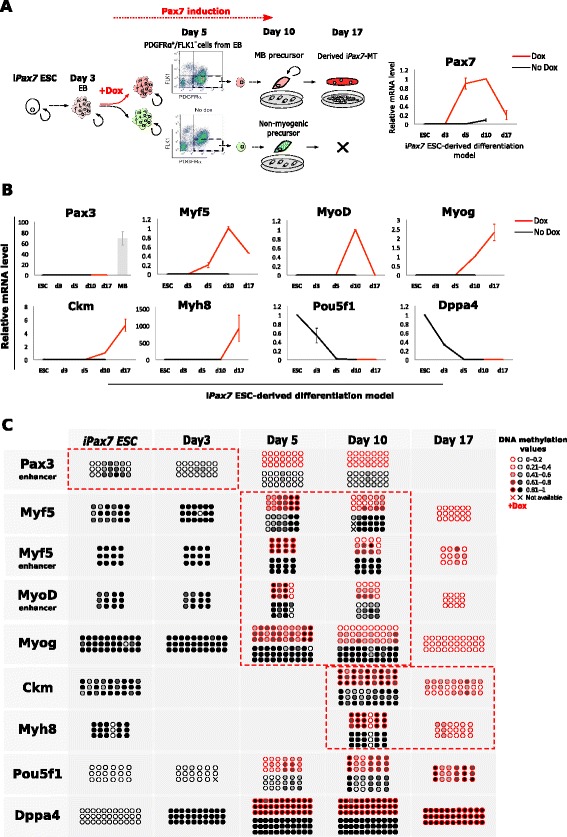
*Pax7*-engineered ESCs efficiently acquired the myogenic DNA methylation profile. **a** Experimental scheme of the differentiation protocol to generate ES-derived myogenic progenitors using i*Pax7* mES cells (*left*). Pax7 induction assessed by qRT-PCR (right). **b** Expression profiles of markers of pluripotency (Pou5f1 and Dppa4), muscle lineage commitment (Pax3, Myf5 and MyoD) and muscle differentiation (Myog, Ckm and Myh8) were measured by qRT-PCR at the successive time points of *iPax7* ESC-derived myogenic model and normalized by *Gapdh* expression (*n* = 3, mean ± SD). **c** DNA methylation dynamics of *Pax3, Myf5* and *MyoD* enhancers and CpG-poor promoters of muscle-specific genes (*Myf5 Myog*, *Ckm* and *Myh8*) and pluripotency genes (*Pou5f1* and *Dppa4*) during *Pax7*-induced myogenesis (+Dox shown in red and -Dox shown in black). The results of three biological replicates are shown. Each *circle* represents a CpG dinucleotide and the colour gradient represents the level of methylation indicated in the legend and assessed by sodium bisulphite sequencing. Demethylation waves occurring during the *Pax7*-induced myogenesis are denoted by *red dotted boxes. ESCs* embryonic stem cells, Dox doxycycline; i*Pax7* inducible *Pax7*

In order to recapitulate the commitment of myogenic precursors during embryonic development, we utilized mouse ES cells engineered to express *Pax7* under the control of a doxycycline-inducible promoter (i*Pax7*) [41]. At day 3 of embryoid body (EB) differentiation *Pax7* transgene expression was induced by doxycycline (Dox), and at day 5 of EB differentiation the mesodermal precursor cells (PDGFRα^+^/FLK1^-^) were sorted, with or without Dox, and grown for 5 more days (Fig. 3a). Importantly, only the i*Pax7*-ES-derived myogenic precursors treated with Dox expressed myogenic differentiation markers such as Myogenin, Ckm and Myh8 and generated myotubes upon differentiation (Fig. 3b and Additional file 3). To assess to what extent the i*Pax7-*ES-derived myogenic progenitors were epigenetically reprogrammed, we evaluated the DNA methylation state of the main myogenic genes in three independent time courses. As expected, preliminary analysis revealed the complete unmethylated state of the CGI located at the *Pax3* and *MyoD* promoter regions during the differentiation process (data not shown). Importantly, the CpG-poor myogenic regulatory regions also showed a very similar DNA methylation signature compared to primary muscle stem cells and, interestingly, a stepwise DNA demethylation was identified (Fig. 3c). The *Pax3* hypaxial enhancer became completely demethylated at day 3 of EB formation, independently of *Pax7* induction and despite the lack of *Pax3* expression at any stage of differentiation (Fig. 3b, c). Contrarily, the -20 kb enhancer of *MyoD*, the -111 kb enhancer of *Myf5* (where Pax7 is bound) and Myf5 promoter became partially demethylated at a later time and exclusively in *Pax7*-induced myogenic precursors, coinciding with the increase in mRNA expression. Notably, at day 17 (i*Pax7*-MTs) the demethylated state persisted, despite the dramatic reduction of *MyoD* and *Myf5* expression levels (Fig. 3b, c). *Myogenin* promoter showed a similar demethylation time course, reaching a total loss of DNA methylation at the i*Pax7*-MB-precursor stage when *Myogenin* was slightly expressed, and maintained in i*Pax7*-MTs, when the *Myogenin* gene showed the maximum expression level (Fig. 3b, c). Interestingly, the promoters of the late differentiation genes *Myh8* and *Ckm* remained fully methylated until the i*Pax7*-MT stage, when they became partially demethylated simultaneously with gene activation (Fig. 3b, c). Importantly, we did not observe any loss of DNA methylation in the absence of Pax7 expression in the control cells (no Dox treatment), indicating that the observed demethylation was skeletal muscle-specific. Finally, the analysis of the promoter regions of the pluripotency genes *Pou5f1* and *Dppa4,* which are progressively silenced, showed a similar gain of DNA methylation during cell differentiation as observed in primary myoblasts (Fig. 3b, c).

### Impaired myogenic differentiation in Apobec2 knockdown myogenic precursors is associated with reduced DNA demethylation

DNA demethylation at specific myogenic regulatory regions was clearly observed in primary myoblasts and in i*Pax7* ESC-derived myogenic precursors (Figs. 1b, 2a and 3c). At first, we questioned whether the reported demethylation could result from a passive loss of methylation, in which the lack or the significant reduction of DNA methyltransferases (Dnmts) coupled to the successive cell divisions would lead to the loss of this epigenetic mark. We analysed the expression levels of the Dnmt1 responsible for the maintenance of the existent DNA methylation pattern, and *Dnmt3a* and *Dnmt3b*, which add *de novo* methyl groups after DNA replication. All Dnmts were expressed, and, as shown in Additional file 4, the expression levels of *Dnmt1* and *Dnmt3a* did not significantly change during EB differentiation, while *Dnmt3b* expression decreased during cell differentiation independently of *Pax7* expression. These observations would suggest that specific DNA methylation reduction on muscle-related genes might be mediated by an active mechanism rather than by a passive one.

**Fig. 4:**
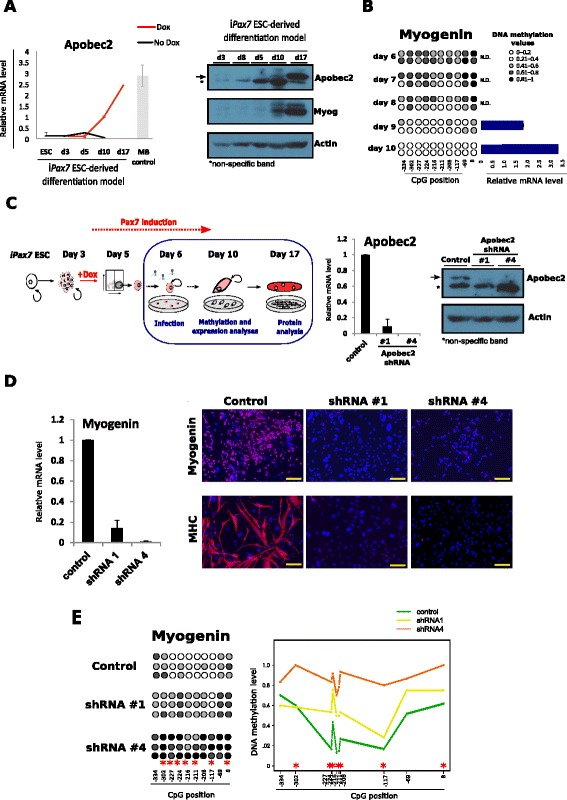
Impaired myogenic progression and differentiation and reduced DNA demethylation in Apobec2 knockdown myogenic precursors. **a** Time course of Apobec2 expression during differentiation of i*Pax7* ES cells in presence or absence of Pax7, which was induced by adding doxycycline (Dox) to the culture media. Apobec2 expression was measured by qRT-PCR (*left*), and by western blotting (WB) (*right*). Apobec2 mRNA expression was normalized by *Gapdh* expression (*n* = 3, mean ± SD) and as a reference the expression level in primary myoblasts (MB control) was measured (*n* = 2, mean ± SD). Myog and actin WB were used as controls for myogenic differentiation and equal loading, respectively. **b** DNA methylation and gene expression analyses of *Myogenin* were performed in a daily time course after Dox induction in the inducible myogenic model (*n* = 2). Each *circle* represents a CpG dinucleotide and its distance to the gene TSS is indicated below. The colour gradient represents the methylation level indicated in the legend. N.D. means non detectable. **c** Scheme of the experimental approach followed to knockdown Apobec2 in the inducible *Pax7* model (*left*). Gene expression and protein level to evaluate Apobec2 knockdown efficiency using two independent clones were compared to control vector (*right*) (*n* = 3, mean ± SD; representative Apobec2 WB with the corresponding actin loading control). **d** *Myogenin* expression was measured in *Pax7*-induced myogenic precursors infected with Apobec2 shRNAs 4 days upon lentiviral transduction (*right*) (*n* = 3, mean ± SD). Representative images of *Pax7*-induced myogenic precursors infected with Apobec2 shRNAs under differentiation conditions stained with Myogenin and MHC antibodies. Scale bar: 100 μm. **e** DNA methylation analysis by sodium bisulphite sequencing of the *Myogenin* promoter in *Pax7* ES-derived myogenic precursors transduced with Apobec2 shRNA1 and shRNA4 in three biological replicates. Each *circle* represents a CpG dinucleotide and its distance to the gene TSS is indicated below. The colour gradient represents the level of methylation indicated in the legend. Statistical significance with respect to the control samples is indicated with * for *p*-value < 0.05 applying the Kruskal-Wallis test. *ESC* embryonic stem cell, *TSS* transcription start site, *shRNA* short hairpin RNA

The two major pathways involved in controlling the DNA demethylation process are the oxidation-mediated demethylation by TET proteins and the cytosine deamination by AID/APOBEC cytidine deaminase enzymes. The expression levels of Tet1, Tet2 and Tet3 did not change in the presence or absence of *Pax7* expression during the EB differentiation, as shown in Additional file 4b. However, the expression levels of Apobec2, the skeletal muscle specific member of the AID/APOBEC family, were highly induced upon Pax7-mediated muscle-commitment, being very high at the progenitor stage and derived MTs (Fig. 4a). Importantly, despite the lack of high specificity of the Apobec2 antibody (Additional file 4c) a clear increase of Apobec2 protein was observed by western-blot concomitant with the differentiation progression (Fig. 4a).

In order to test whether Apobec2 is required for the myogenic process, we performed loss of function experiments by down-regulating *Apobec2* levels using lentiviral vectors encoding for specific shRNAs. To identify the most appropriate time point for shRNA viral-mediated transduction (before DNA demethylation has taken place) we performed and analysed a daily time course starting from Dox-treated PDGFRα^+^/FLK1^-^ sorted cells from day 5 EBs until i*Pax7*-MB precursor stage (day 10). As shown in Fig. 4b, we observed a perfect correlation between gradual DNA demethylation of the *Myogenin* locus and increased gene expression, which starts at EB day 9. Based on these data, we decided to transduce Dox-treated PDGFRα^+^/FLK1^-^ cells at day 6 of differentiation using two different Apobec2 shRNAs (shRNA1 and shRNA4). As expected, four days after transduction, Apobec2 shRNAs dramatically knocked-down Apobec2 mRNA and protein levels (Fig. 4c) without affecting *Pax7* expression (data not shown). Importantly, *Apobec2* knockdown was associated with a severe down-regulation of *Myogenin* expression, both at mRNA and protein level and, ultimately, impairment of terminal muscle differentiation (Fig. 4d). Interestingly, the methylation analysis of the *Myogenin* promoter upon *Apobec2* knockdown showed a significant methylation gain in 70 % of the promoter CpGs (Kruskal-Wallis test, *p*-value < 0.05) compared to the control cells (Fig. 4e). Finally, we also analysed the impact of *Apobec2* knockdown in *MyoD* and *Myf5* DNA methylation levels, and, as shown in Additional file 5a,b, although we observed a small increase of methylation in some of the CpGs, it was not statistically significant. All these results together, suggest that Apobec2 might participate more actively in the DNA demethylation process required to drive myogenic differentiation rather than in regulating the DNA methylation-dependent myogenic commitment.

## Discussion

Tissue-specific stem cells are lineage-restricted cells that have lost pluripotency capacities and gained cell-type identity. Epigenetic regulation, including DNA methylation and the post-translational modifications of histones, is crucial in the establishment and maintenance of cellular identity [1–3, 42]. In this study, we analysed for the first time the DNA methylation dynamics of the principal genes orchestrating myogenic determination and differentiation programs by comparing ES cells and their *Pax7*-induced skeletal myogenic derivatives with muscle stem cells, both in proliferating and differentiating conditions. The detailed analysis of CpG-poor regulatory regions for the developmental gene *Pax3, MyoD* and *Myogenin* genes as muscle cell-identity transcription factors, and *Ckm*, *Mhy1*, *Myh4* and *Myh8* as myogenic terminal differentiation genes revealed a specific DNA demethylation signature required to acquire and maintain muscle-cell identity during the establishment of the muscle lineage (Figs. 1b, 2a and 3c). These results reinforce observations reported in other cellular models that lineage-specific DNA demethylation, together with pluripotency genes hypermethylation, occur during early cell fate decisions conferring unique DNA methylation patterns which correlate with defined cellular identities [43–46]. Interestingly, our results showed that the crucial myogenic differentiation factor Myogenin became fully demethylated in muscle-committed cells before gene expression (Fig. 2a). However, these results were in disagreement with previous work performed in C2C12 cells where loss of methylation was observed between myoblasts and myotubes [47, 48]. To address this controversy, we isolated freshly satellite cells from adult mice, and, interestingly, the DNA methylation analysis of the *Myogenin* promoter revealed the complete demethylation of the promoter in ex vivo isolated muscle stem cells (unpublished data). These results would indicate that DNA demethylation of *Myogenin* promoter occurs during muscle-lineage determination, allowing the acquisition of a transcriptional poised state before gene expression during muscle differentiation. Notably, gene silencing of terminal differentiation *Myosin* genes in proliferating myoblasts upon DNA demethylation is maintained by the acquisition of the Polycomb repressive mark H3K27me3 (Fig. 2b), as previously reported for *Myog* and *Ckm* promoters [49, 50]. During terminal differentiation, H3K27me3 is replaced by the positive marks H3K4me3 and H3K27Ac, which result in gene transcription. This two-step repressive mechanism, where DNA methylation is replaced by H3K27me3, is also observed when ESCs are induced to differentiate [51] and represents a switch from a stable repression mediated by DNA methylation to a more facultative repression mediated by the histone code [4].

*Mrf4* is considered a determination and differentiation myogenic gene and is the only MRF expressed in mature myofibres [52, 53]. Curiously, *Mrf4* promoter is the only one still repressed by DNA methylation in undifferentiated myoblasts, being highly demethylated only in mature myofibres where it is highly expressed (Fig. 2a). A recent paper demonstrated that adult satellite cells emerged from embryonic founder cells in which the *Mrf4* gene was activated [54], but intriguingly, our results showed that *Mrf4* promoter was completely methylated in all cell types analysed except in mature myofibres. Very recently we demonstrated that the multiple enhancers regulating the complex spatial-temporal expression of the *Myf5/Mrf4* locus during embryogenesis were specifically demethylated in muscle-committed cells [16]. In that context, we speculate that complete demethylation of *Mrf4* promoter would be only required in mature myofibres to fully activate and maintain its high expression levels.

**Fig. 5:**
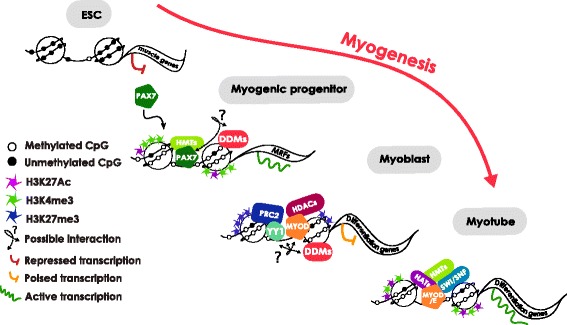
Model for the epigenetic control of muscle-specific gene expression during myogenesis. Diagram proposing a simplified model where myogenic transcription factors would activate muscle genes by modulating both the recruitment, direct or indirect, of DNA demethylases to reduce DNA methylation levels and chromatin compaction, and histone modifiers/chromatin remodelers to activate transcription of muscle genes. In proliferating myoblasts, the differentiation genes would be kept in a silenced/poised transcriptional state by PRC2 and HDACs complexes, whereas in myotubes they would be expressed upon the recruitment of chromatin activating complexes. Abbreviations: *DDMs* DNA demethylases, *HATs* histone acetyltransferases, *HDACs* histone deacetylases, *HMTs* histone methyltransferases, *PRC2* Polycomb repressive complex 2, *SWI/SNF* SWI/SNF ATP-dependent chromatin remodelling complex, *YY1* Yin and Yang 1

It is well known that Pax7 has a key role in the formation and maintenance of muscle committed progenitors. The engineered i*Pax7*-ESC model let us recapitulate the commitment of myogenic precursors during embryonic development and revealed the acquisition of a very similar DNA methylation signature, compared to primary muscle cells (Fig. 3c). Importantly, these results support the use of i*Pax7* ES-derived myogenic progenitors as a *bona fide* model to generate in vitro myogenic precursors with therapeutic purposes. The detailed analysis of this stepwise model showed that the myogenic differentiation process occurred coupled to a gradual loss of DNA methylation in myogenic regulatory regions in consecutive waves. Initially, *Pax3* enhancer became demethylated before *Pax7* induction in line with its expression pattern, which is not restricted to the myogenic lineage [55]. Next, *Myf5*, *MyoD* and *Myogenin* CpG-poor regulatory regions became demethylated in i*Pax7*-myoblast precursors correlating with transcriptional activation (Fig. 3b-c). Finally, the terminal differentiation myogenic genes *Ckm* and *Myh8* became simultaneously demethylated and activated during late differentiation, when i*Pax7* myogenic precursors differentiated and fused forming myotubes. This late demethylation wave only occurred in ES-derived differentiating myocytes and not in primary myotubes, already demethylated in the MB stage. This observation points out an epigenetically accentuated myogenic commitment of adult muscle stem cells compared to myogenic precursors. Importantly, the loss of DNA methylation only took place upon *Pax7* expression (+Dox) indicating that Pax7 is required for skeletal muscle-specific demethylation. Notably, the differentially methylated enhancer located at -111 kb of *Myf5* TSS is a direct target of Pax7 in satellite cells [56], suggesting a link between Pax7 and DNA methylation. In the last years, several studies have shown the crosstalk between muscle transcription factors and chromatin modifiers. In this regard Pax7 has been shown to recruit the Wdr5-Ash2L-MLL2 histone methyltransferase complex to target genes, in order to stimulate myogenic transcription [57]. In the same line, MyoD interacts with the chromatin remodelling complex SWI/SNF [58, 59], and recruits the histone acetyltransferase P300 and the histone methyltransferase Ash2L to activate muscle genes [60–62]. In that context and taking into account our results we propose a model (Fig. 5) where DNA methylation prevents inappropriate expression of muscle genes in ESCs, being removed during the commitment to the skeletal muscle lineage. The expression of myogenic transcription factors might modulate both the recruitment, direct or indirect, of DNA demethylases to reduce DNA methylation and chromatin compaction, and histone modifiers/chromatin remodelers to activate transcription of muscle genes. In proliferating myoblasts, the differentiation genes would be kept in a silenced/poised transcriptional state by PRC2 and HDACs complexes, whereas in myotubes they would be expressed upon the recruitment of chromatin activating complexes.

To investigate the possible pathway involved in DNA demethylation during myogenesis we focused on Apobec2 (Apolipoprotein B mRNA editing enzyme catalytic polypeptide 2), a member of the cytidine deaminase family expressed exclusively in cardiac and skeletal muscle tissues with unknown functions [63, 64]. The generation of Apobec2^-/-^ mice showed no major defects in mouse health, fertility or survival up to 1 year of age [65]. However, detailed analysis of these animals revealed a 15–20 % reduction in body mass from birth onwards, a clear histological evidence of a mild myopathy during aging, and a markedly increased ratio of slow to fast fibres in *soleus* Apobec2^-/-^ muscles [66]. Although the cytidine deaminase activity of Apobec2 is still controversial [64, 67–69], it has been reported that during zebrafish development Apobec2b stimulates DNA demethylation [70], and AID/Apobec proteins promote demethylation of 5hmCs in mammals [21]. Our results showed that *Apobec2* expression was highly induced upon muscle-commitment in i*Pax7-*myogenic precursors (Fig. 4a) and, importantly, the downregulation of Apobec2 dramatically impaired muscle differentiation, abolishing myogenin and myosins expression (Fig. 4d), in agreement with experiments performed on C2C12 myoblast cells showing that Apobec2 was required for myotube formation [71]. Recently, Powell and co-workers reported that Apobec2 had an essential role during retina and optic nerve regeneration [72], although the small changes observed in DNA methylation during tissue-regeneration were independent of Apobec2 expression [73]. Interestingly, our results showed that *Myogenin* DNA methylation levels were significantly higher in i*Pax7*-myoblast precursors when Apobec2 activity was abolished, correlating with gene silencing (Fig. 4e). However, the *MyoD* and *Myf5* DNA methylation changes observed upon Apobec2 knockdown were smaller, and not statistically significant. That could be due to the existence of some cell heterogeneity during the process of Pax7-dependent myogenic induction, which could mask the demethylation events occurring in *MyoD* and *Myf5* genes. Another explanation could be related to the fact that MyoD and Myf5 play a very early role in muscle-lineage determination, as mouse embryos with targeted mutation in both genes lack myoblasts and differentiated skeletal muscles [74]. Therefore, we cannot rule out the involvement of other mechanisms, such us Tet-mediated DNA demethylation, in the epigenetic activation of these two early-induced genes. Altogether, our results suggest that Apobec2 might participate in the active DNA demethylation process required to drive myogenic differentiation. Apobec2 has emerged as an important myogenic regulator that would merit further studies regarding how it is recruited to genomic loci affecting epigenetic regulation.

## Conclusions

This study presents for the first time the DNA methylation dynamics of the principal genes orchestrating the myogenic determination and differentiation programs during myogenesis. Our results show that a common DNA demethylation signature is required to acquire and maintain the muscle-cell identity, and that downregulation of the muscle-specific cytidine deaminase Apobec2 in ES-derived myogenic precursors reduces myogenic-associated DNA demethylation and impairs muscle differentiation. Importantly, we have also identified muscle epigenetic markers that may be useful to ensure the efficient and safe generation of ES- and iPS-derived myogenic progenitors for therapeutic applications.

## Methods

### Skeletal muscle stem cells isolation and culture

Skeletal muscle stem cells were isolated from hind limb muscles of 6- to 8-week-old mice (kindly provided by P. Muñoz Cánoves of the Pompeu Fabra University, Barcelona) as described in [75]. After mechanical and enzymatic dissociation with 1 % Pronase protease (Calbiochem), the filtered digest was centrifuged through an isotonic Percoll (Amersham) gradient (60 % overlaid with 20 %) and cells were collected from the gradient interface, resuspended in growth medium (GM: Ham’s F-10 plus 20 % FBS, 5 ng/ml bFGF, 100 U/ml penicillin and 100 μg/ml streptomycin) and grown on tissue culture dishes coated with 0.05 mg/ml collagen I from rat tail (Becton and Dickinson) to amplify the myoblast population. To induce myotube formation, confluent proliferating primary myoblasts were grown on matrigel coated culture dishes (Basement Membrane Matrix, Becton and Dickinson) and switched to differentiation medium the next day (DM: DMEM plus 2 % horse serum, 100 U/ml penicillin and100 μg/ml streptomycin (Life Technologies)) for 4 days. Myofibres directly isolated from EDL muscles were kindly provided by S. Gutarra. Two biological replicates of each experiment were performed and analysed. Mouse protocols were approved by the Animal Care and Use Committee of the PRBB, and the Ethical Committee for Animal Experimentation of the Government of Catalonia.

### Cell lines

ESCs were kindly provided by M. Carrió (cGR8) and F. Lluís (E14Tg2), NPCs were also provided by F. Lluís and HL1 cells by S. Pagans. MEFs and HEK 293 T cells were cultured in DMEM complemented with 10 % fetal bovine serum, 1 % penicillin/streptomycin and 2 mM Glutamax (Life Technologies).

### Inducible *Pax7*-ESC-derived myogenic model

The inducible cell line encoding full length murine Pax7 (i*Pax7*) was generated as previously described in [41]. i*Pax7*-ESCs were co-cultured with irradiated mouse embryonic fibroblasts (MEFs), obtained as described in [76]. The co-culture was maintained in knockout DMEM (Invitrogen) supplemented with 15 % ES qualified FBS (Invitrogen), 1 % penicillin/streptomycin (Invitrogen), 2 mM Glutamax, 0.1 mM b-mercaptoethanol and 1000 U/ml Lif (Millipore) medium. To induce cellular differentiation ESCs were trypsinized, resuspended in EB differentiation medium (EBM) containing Iscove-modified DMEM (Life Technologies), 15 % ES qualified FBS (Life Technologies), 1 % penicillin/streptomycin, 2 mM Glutamax, 200 mg/ml iron-saturated transferrin (Sigma-Aldrich), 4.5 mM monothioglycerol (Sigma-Aldrich, and 50 mg/ml ascorbic acid (Sigma-Aldrich), and preplated for 30 min on gelatin-coated dishes to remove MEFs. After counting, 1x10^6^ cells were plated in 15 cm petri dishes and placed on a shaker at 60 RPM/min (37 °C, 5 % CO_2_) to induce EB formation (this time point is referred to as day 0 of the EB differentiation protocol). *Pax7* expression was induced after 3 days of differentiation by adding doxycycline (Dox) (D9891, Sigma-Aldrich) to the culture medium at a final concentration of 1 μg/ml. At day 5, mesodermal precursors corresponding to the PDGFRα^+^/FLK1^-^ cell population were isolated by FACS (FACS Aria II - BD Biosciences) using PE-conjugated anti-mouse CD140a (PDGFRα; RRID: AB_657615) and APC-conjugated anti-mouse CD309 (FLK1; RRID: AB_657865) (E-Bioscience). Sorted cells from Dox-treated and untreated EBs were cultured on gelatin-coated flasks in EBM (with or without Dox) for 5 more days before harvesting them (which we refer to as i*Pax7* myoblasts (MB) precursor cells). To induce the myotube formation, the i*Pax7*-MB precursors were cultured on DMEM (Invitrogen) supplemented with 2 % horse serum (Hyclone), 1 % penicillin/streptomycin and 2 mM glutamax and, importantly, Dox was removed to allow terminal myogenic differentiation, which physiologically only occurs upon *Pax7* expression withdrawal.

### Lentiviral transduction

*Apobec2* shRNAs cloned in pLKO.1-puro lentiviral constructs were purchased by the Biomedical Genomic Center at the University of Minnesota. After testing different clones for *Apobec2* down-regulation compared to the control vector, the following clones were selected: shRNA#1 CCTGGCTTCCTGATTCTACTT and shRNA#4 GCTACCAGTCAACTTCTTCAA. Lentiviruses were produced by co-transfecting pLKO.1-puromycine vector and the packaging constructs (pVSV-G, pREV and pD8.74 [77]) in HEK 293 T cells using Lipofectamine LTX-Plus Reagent (Life Technologies) following the manufacturer’s instruction. Supernatants containing the lentiviral particles were collected 36 h after transfection, passed through a 0.45 μm filter and used to transduce i*Pax7* MB precursors. Cells were harvested for gene expression and DNA methylation analyses 4 days after transduction.

### Gene expression analysis

Total RNA was isolated with a miRNeasy Mini Kit (Qiagen) according to the manufacturer’s instructions and retro-transcribed with SuperScript® III Reverse Transcriptase (Life Technologies). cDNA was amplified by qRT-PCR using LightCycler480 (Roche) with Fast Start DNA Master Sybr Green I mix (Roche) and expression results were normalized with *Gapdh* or *18S* expression. Expression results for the Pax7-induced ES-derived muscle differentiation model were plotted relative to i*Pax7* myogenic precursors (set to 1) for myogenic markers and to i*Pax7*-ESC for pluripotency markers. Primer sequences are listed in Additional file 6.

### Immunofluorescence staining

Cells cultured on slides were fixed with 4 % paraformaldehyde for 10 min, permeabilized with 0.1 % Triton X-100 (Life Technologies) for 10 min and blocked with 5 % BSA reagent (Vector Labs) for 30 min, before incubating with primary antibodies Myogenin (1:250, clone F5D, BD Biosciences; RRID: AB_39638) and MHC (1:20, clone MF20, Developmental Studies Hybridoma Bank; RRID: AB_2147781). Alexafluor 555-goat anti-mouse (1:500, Invitrogen; RRID: AB_2535844) was used as secondary antibody and preparations were mounted with Prolong mounting media with DAPI (Life Technologies) to counter stain nuclei.

### Sodium bisulphite conversion, sequencing and pyrosequencing validation

Genomic DNA was isolated after an overnight Proteinase K digestion (Invitrogen) followed by an RNaseA (Invitrogen) treatment for 1 h. After phenol-chloroform extraction, ethanol precipitation and NanoDrop quantification, genomic DNA was run in a 1 % agarose gel stained with ethidium bromide to check its integrity. Sodium bisulphite conversion was performed using 300 ng of genomic DNA with an EZ DNA Methylation-Gold™ Kit (ZymoResearch, Orange) according to the manufacturer’s instructions. Treated DNA was amplified as previously described [78], purified and sequenced by Sanger direct sequencing. In Additional file 7 the specific primers for the methylation profiling of the different genomic regions are listed. In Additional file 8 it is shown how the DNA methylation range was quantified and the corresponding validation by pyrosequencing. Briefly, to assess the DNA methylation state of each cytosine we analysed the raw sequencing electropherograms visualized with Geospiza FinchTV software. We compared the DNA reference sequence with the obtained sequence after bisulphite conversion. In bisulphite-treated DNA followed by PCR amplification the methylated cytosines remain cytosines (C, blue peak in the sequencing electropherogram), whereas the unmethylated cytosines become thymines (T, red peak in the sequencing electropherogram). Notice that when reverse sequencing primers are used the cytosines will be read as guanines (G, black peak) and the thymines as adenines (A, green peak). When a single peak was observed in the original C position, we considered the population homogenous, indicating a blue peak (C) that the cytosine was methylated in all cells and a red peak (T) that the cytosine was unmethylated in all cells. When two peaks were shown in the original C position it corresponded to a mixed population. In these cases, the height of each peak was measured to assess the proportion of each population. For the sake of simplicity, the methylation level was ranked in five different intervals 0–0.2, 0.21–0.4, 0.41–0.6, 0.61–0.8 and 0.81–1, which reflected the DNA methylation state of this particular cytosine. In Additional file 8b the pyrosequencing validation of the methylation state of three CpGs located in Myh1 and Myh8 promoter regions is shown. Notably, the linear correlation between the results obtained by both methodologies was R^2^ = 0.98 for Myh1 and R^2^ = 0.91 for Myh8.

### Western blot analysis

Proteins were extracted using RIPA buffer supplemented with protease inhibitors (Complete Mini, Roche) and quantified using Bradford reagent (Sigma). An amount of 50 ug of proteins were separated on a 12 % SDS-PAGE gel and successively transferred on PVDF membrane (Millipore). The membrane was blocked using 5 % BSA in TBST and then incubated overnight with the indicated antibody diluted in blocking solution. Antibodies used were: anti-Apobec2 (1:1000, sc-98335 Santa Cruz Biotechnology; RRID: AB_2258415), anti-MyoG (1:1000, clone F5D Developmental Studies Hybridoma Bank; RRID: AB_2146602), and anti-Actin (1:2000, MAB1501 Millipore; RRID: AB_2223041). HRP-conjugated anti-mouse or anti-rabbit IgG (1:20000, Amersham ECL Western Blotting Reagent Pack RPN2124) were used as secondary antibodies. Positive and negative controls used for the identification of Apobec2 consisted of skeletal muscle and brain protein extracts, respectively.

### Availability of data and materials

All genomic representations containing reference genes, CpG islands and ChIP-seq data were integrated, explored and visualized using the Integrative Genomics Viewer [79]. All ChIP-seq data, with the exception of the H3K27Ac and p300 data from proliferating and differentiated C2C12 (MB and MT) [38], were generated by the ENCODE Project Consortium [37]. GEO accession numbers are listed in Additional file 9. Sodium bisulphite sequencing data and Kruskal-Wallis test of sodium bisulphite data were done with the Methylation Plotter web tool [80]. Supporting data containing the individual gene expression values are shown in Additional file 10.

## Additional files

Additional file 1: CpG-island promoters of developmental genes are unmethylated. DNA methylation state of CpG islands overlapping and surrounding the promoter region of *Pax3* (a) and *Pax7* (b) genes in myogenic (MB, MT, MF) and non-myogenic samples (ESC). CpG islands are indicated in green and regions analysed by sodium bisulphite sequencing are shown in red. Each circle represents a CpG dinucleotide and its distance to the gene TSS is indicated below. The colour gradient represents the percentage of methylation indicated in the legend. Abbreviations: ESC, embryonic stem cell; MB, myoblast; MT, myotube; MF, myofiber; TSS, transcription start site. c. DNA methylation state of -5 kb distal regulatory region for MyoD was analysed by sodium bisulphite sequencing in ESC and myoblast samples, and represented as above. (PDF 171 kb) Additional file 2: CpG-poor promoters of pluripotency genes became methylated during muscle-lineage specification. a. Scheme of the Sox2 and Fgf4 loci showing in dark green the CpG island and in light green the unmethylated state of the analysed CpGs by HAIB-Methyl RRBS from ENCODE Project in human ESCs (H1-ESCs), myoblasts (HSMM), myotubes (HSMMtube) and myofibers (skeletal_7N). b. DNA methylation state of *Pou5f1* (left) and *Dppa4* (right) promoters in myogenic (MB, MT, MF) and non-myogenic samples (ESC, NPC, HL1, MEF). Analysed regions are indicated in red and biological duplicates are shown in the circle charts, where each circle represents a CpG dinucleotide and its distance to the gene TSS is indicated below. The colour gradient represents the percentage of methylation indicated in the legend. Bar charts show the gene expression values measured by qRT-PCR and normalized by *Gapdh* and *18S*, respectively (n = 2, mean ± SD). N.D. means non detectable. c. Histone marks distribution and p300 binding in ESC (blue), MB (yellow) and MT (red) obtained from ENCODE Project and Dynlatch’s lab [38]. Abbreviations: ESC, embryonic stem cell; HL1, cardiomyocyte; MB, myoblast; MT, myotube; MF, myofiber; MEF, mouse embryonic fibroblast; NPC, neuronal precursor cell; TSS, transcription start site. (PDF 153 kb) Additional file 3:
*Pax7*-induced ESC-derived myogenic model. Representative fields showing the morphology of i*Pax7* ES-derived cells at different stages of differentiation with or without Pax7 induction (100x magnification). Arrows show plurinucleated myotubes. ESCs, MB and MT precursors were cultured in monolayer, whereas EBs day 3 were grown in suspension. Abbreviations: EBs, embryoid bodies; ESCs, embryonic stem cells; MB, myoblast; MT, myotube; i*Pax7*, inducible *Pax7*; Dox, Doxycycline. (PDF 25026 kb) Additional file 4: Dnmt and Tet expression profiles during *Pax7*-induced ESC derived myogenic model and Apobec2 western blot analysis. Expression levels of Dnmt1, Dnmt3a and Dnmt3b (a) and Tet1, Tet2 and Tet3 (b) were measured by qRT-PCR at the successive time points of i*Pax7* ESC-derived myogenic model, with or without doxycycline, and normalized by *Gapdh* expression (*n* = 3, mean ± SD). c. Apobec2 western blots were performed on total protein extracts from skeletal muscle tissue (SK M) and brain (left), and muscle progenitor cells (i*Pax7*-MB) and mature myotubes (C2C12-MT) (right). Skeletal muscle shows a specific Apobec2 band (32 kDa), whereas multiple bands are detected in brain tissue and in muscle cells. (PDF 798 kb) Additional file 5: DNA methylation analysis of *MyoD* and *Myf5* genes in Apobec2 depleted cells. DNA methylation analysis by sodium bisulphite sequencing of the -20 kb *MyoD* enhancer (a) and promoter and -111 kb enhancer of *Myf5* gene (b) in *Pax7* ES-derived myogenic precursors transduced with Apobec2 shRNA1 and shRNA4, in three biological replicates. Each circle represents a CpG dinucleotide and its distance to the gene TSS is indicated below. The colour gradient represents the methylation level indicated in the legend. (PDF 100 kb) Additional file 6: Primer sequences used by qRT-PCR expression analysis. (DOCX 12 kb) Additional file 7: Primer sequences used by assessing the DNA methylation profiles of different genomic regions either by Sanger sequencing or pyrosequencing. (DOCX 16 kb) Additional file 8: Explanation and validation of the Sanger sequencing-based DNA methylation method. a. Quantification of DNA methylation state by bisulphite conversion followed by Sanger direct sequencing. DNA methylation state of each cytosine was analysed comparing the DNA reference sequence with the raw sequencing electropherograms of the bisulphite-converted sequence, visualized with Geospiza FinchTV software. The methylation level was ranked in five different intervals 0–0.2, 0.21–0.4, 0.41–0.6, 0.61–0.8 and 0.81–1, which reflects the DNA methylation state of this particular cytosine. Detailed explanations about how the methylation interval was assigned for each cytosine are in the Methods section. b. Correlation between Sanger and Pyrosequencing results. In the left panels are shown the DNA methylation state of three CpGs located in Myh1 and Myh8 promoter regions, in two embryonic stem cells ESC 1-2 and two primary myogenic precursor cells MPC 1-2. For each CpG it is shown the raw chromatogram obtained by Sanger sequencing with our corresponding assigned methylation interval and the methylation value obtained by pyrosequencing, which is inside the interval in most of the cases (right top scatter plots). On the right bottom scatter plots are shown the high linear correlation between Sanger sequencing and pyrosequencing results, with R^2^ = 0,98 for Myh1 and R^2^ = 0,91 for Myh8. (PDF 2930 kb) Additional file 9: GEO accession numbers for histone marks ChIP-seq data generated by the ENCODE Project Consortium and Dynlacht’s laboratory. (DOCX 43 kb) Additional file 10: Individual gene expression values normalized by a reference gene are shown for the two or three biological replicates analysed. (XLSX 49 kb)

## Abbreviations

CGI: CpG island
Dox: Doxycycline
EB: Embryoid body
ESCs: Embryonic stem cells
i*Pax7*: Inducible *Pax7*
MB: Myoblasts
MF: Myofibres
MT: Myotubes
