# Supplementary figures and images for: Muscle cell identity requires Pax7-mediated lineage-specific DNA demethylation
Source: BMC Biol. 2016 Apr 13;14:30. doi: 10.1186/s12915-016-0250-9 (PMC4831197; doi:10.1186/s12915-016-0250-9)

**A**

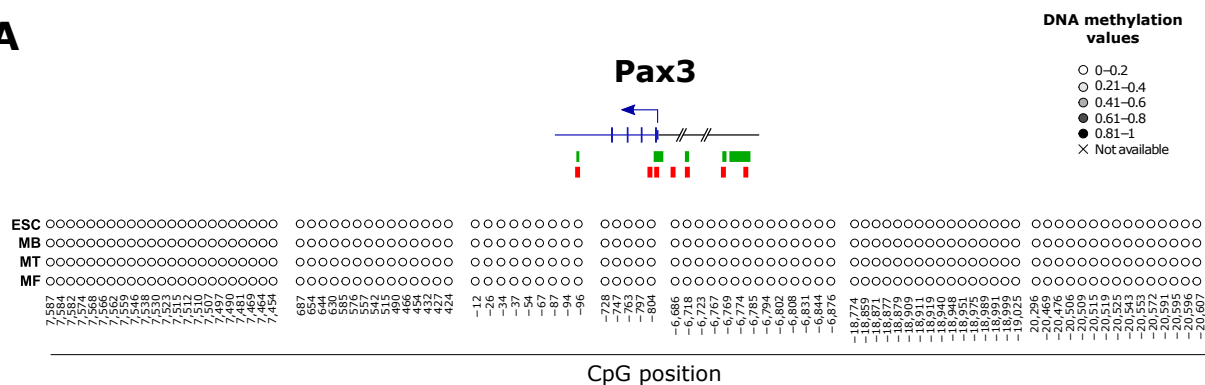

# B

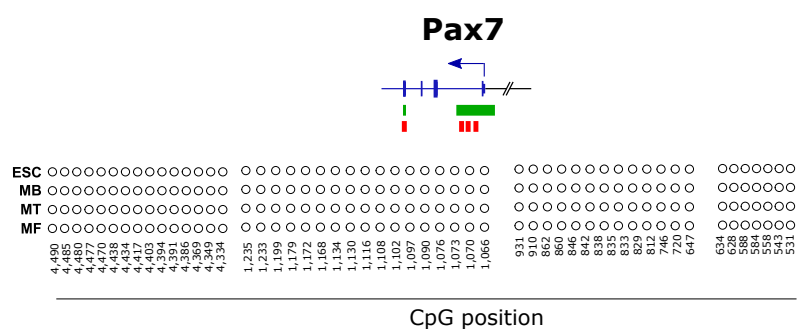

**C**

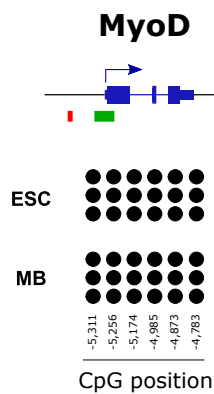

Supplement: Additional file 1: — CpG-island promoters of developmental genes are unmethylated. DNA methylation state of CpG islands overlapping and surrounding the promoter region of Pax3 (a) and Pax7 (b) genes in myogenic (MB, MT, MF) and non-myogenic samples (ESC). CpG islands are indicated in green and regions analysed by sodium bisulphite sequencing are shown in red. Each circle represents a CpG dinucleotide and its distance to the gene TSS is indicated below. The colour gradient represents the percentage of methylation indicated in the legend. Abbreviations: ESC, embryonic stem cell; MB, myoblast; MT, myotube; MF, myofiber; TSS, transcription start site. c. DNA methylation state of -5 kb distal regulatory region for MyoD was analysed by sodium bisulphite sequencing in ESC and myoblast samples, and represented as above. (PDF 171 kb) [file 12915_2016_250_MOESM1_ESM.pdf]

A

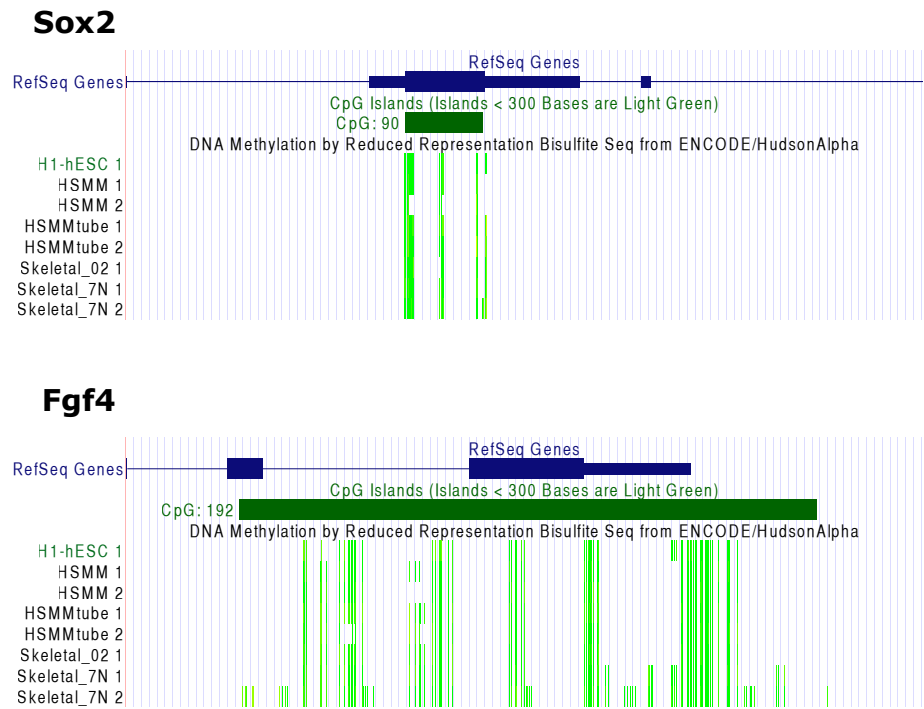

B

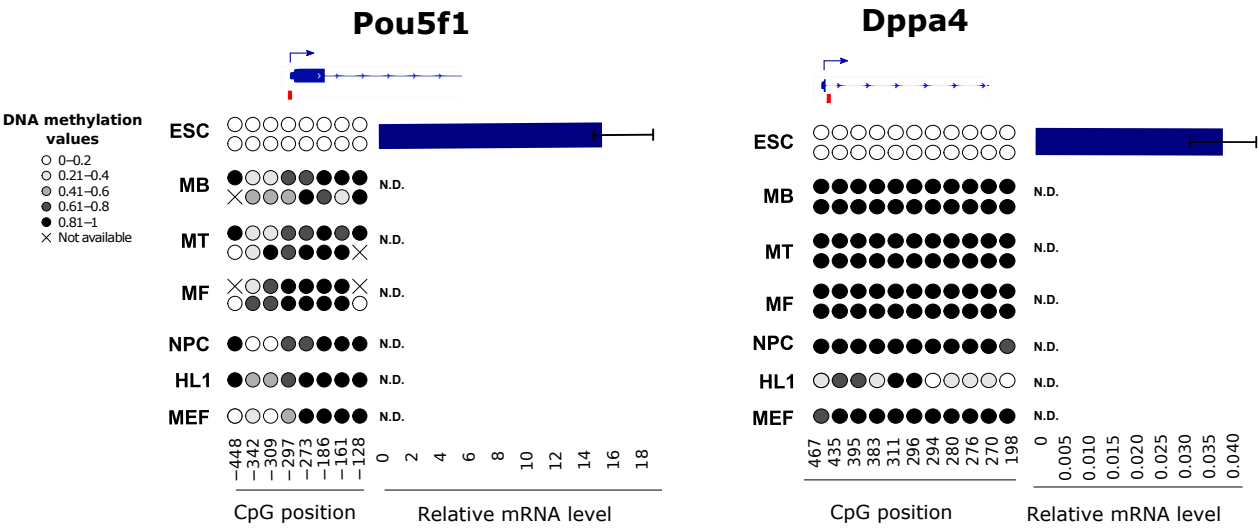

C

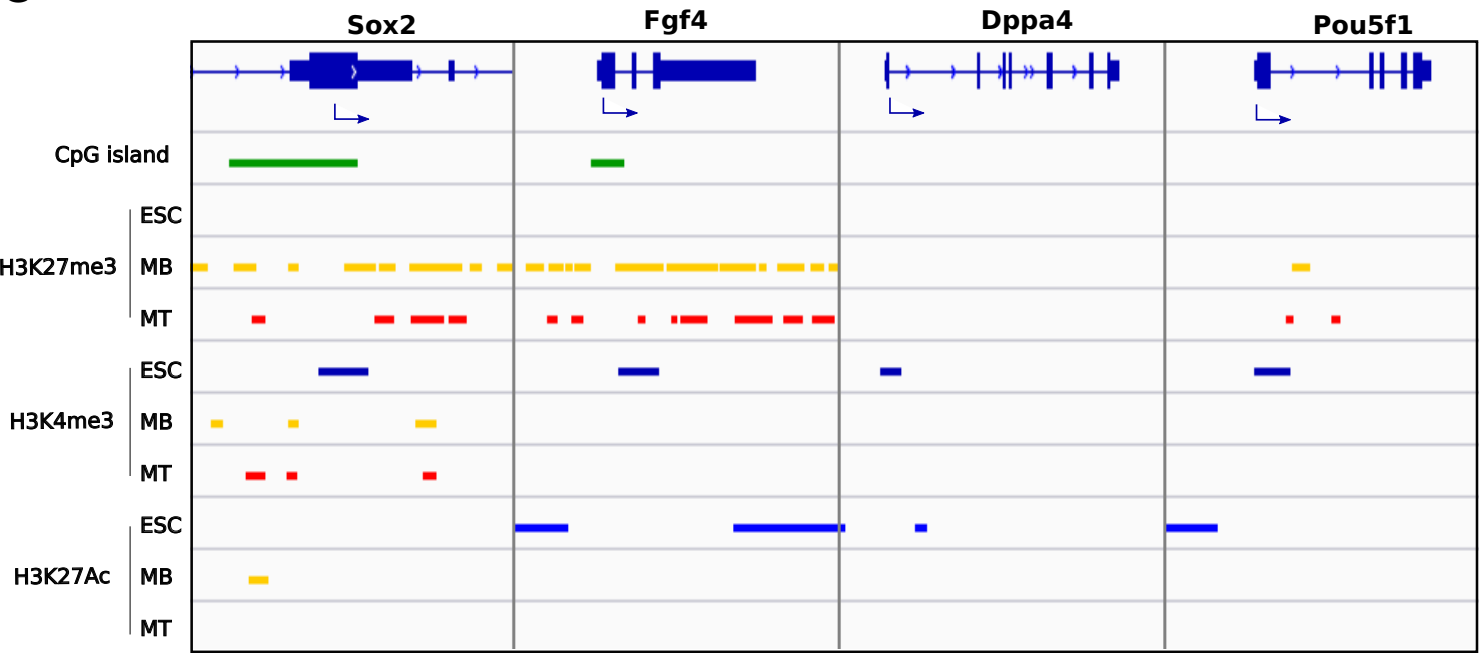

Supplement: Additional file 2: — CpG-poor promoters of pluripotency genes became methylated during muscle-lineage specification. a. Scheme of the Sox2 and Fgf4 loci showing in dark green the CpG island and in light green the unmethylated state of the analysed CpGs by HAIB-Methyl RRBS from ENCODE Project in human ESCs (H1-ESCs), myoblasts (HSMM), myotubes (HSMMtube) and myofibers (skeletal_7N). b. DNA methylation state of Pou5f1 (left) and Dppa4 (right) promoters in myogenic (MB, MT, MF) and non-myogenic samples (ESC, NPC, HL1, MEF). Analysed regions are indicated in red and biological duplicates are shown in the circle charts, where each circle represents a CpG dinucleotide and its distance to the gene TSS is indicated below. The colour gradient represents the percentage of methylation indicated in the legend. Bar charts show the gene expression values measured by qRT-PCR and normalized by Gapdh and 18S, respectively (n = 2, mean ± SD). N.D. means non detectable. c. Histone marks distribution and p300 binding in ESC (blue), MB (yellow) and MT (red) obtained from ENCODE Project and Dynlatch’s lab [38]. Abbreviations: ESC, embryonic stem cell; HL1, cardiomyocyte; MB, myoblast; MT, myotube; MF, myofiber; MEF, mouse embryonic fibroblast; NPC, neuronal precursor cell; TSS, transcription start site. (PDF 153 kb) [file 12915_2016_250_MOESM2_ESM.pdf]

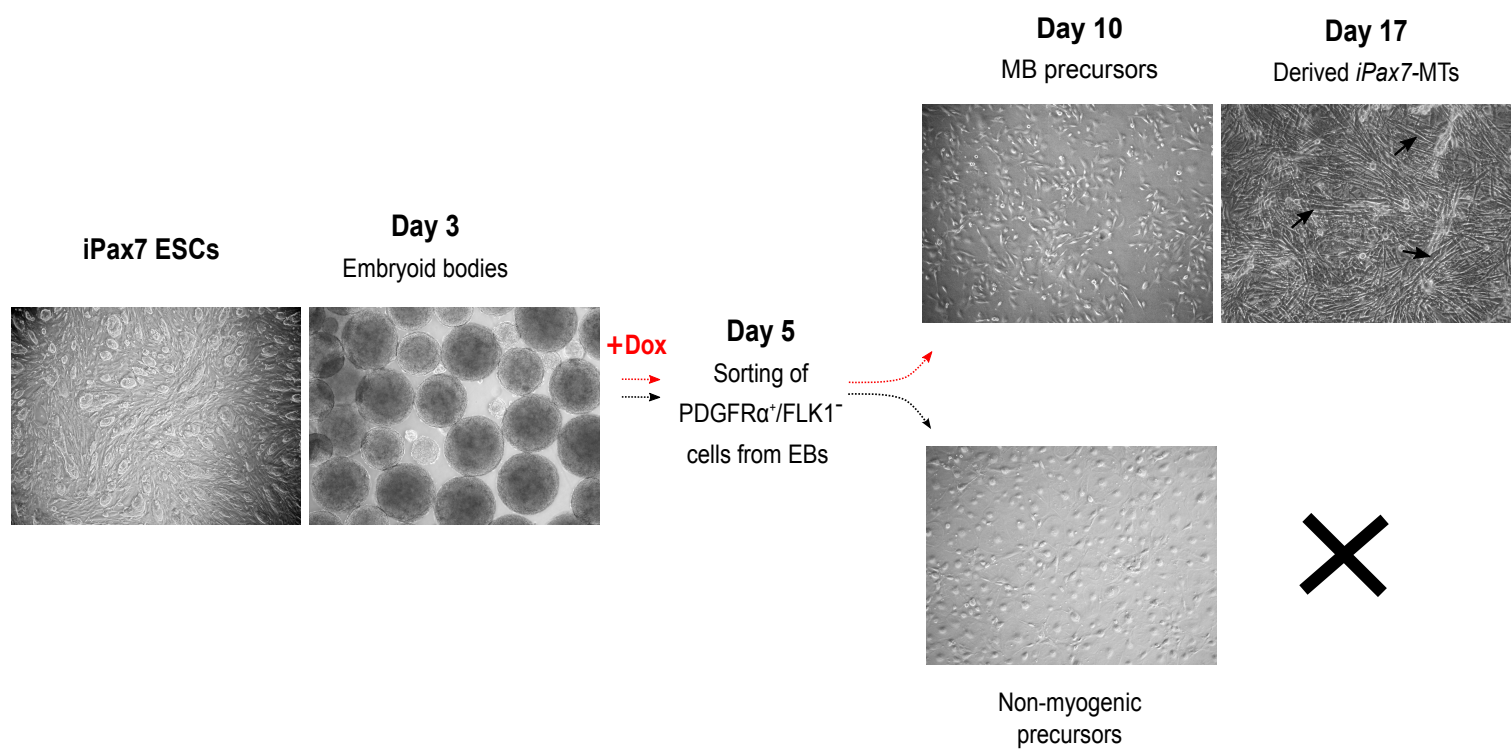

**Supp. Figure 3**  
**Carrió et al.**

Supplement: Additional file 3: — Pax7-induced ESC-derived myogenic model. Representative fields showing the morphology of iPax7 ES-derived cells at different stages of differentiation with or without Pax7 induction (100x magnification). Arrows show plurinucleated myotubes. ESCs, MB and MT precursors were cultured in monolayer, whereas EBs day 3 were grown in suspension. Abbreviations: EBs, embryoid bodies; ESCs, embryonic stem cells; MB, myoblast; MT, myotube; iPax7, inducible Pax7; Dox, Doxycycline. (PDF 25026 kb) [file 12915_2016_250_MOESM3_ESM.pdf]

**A**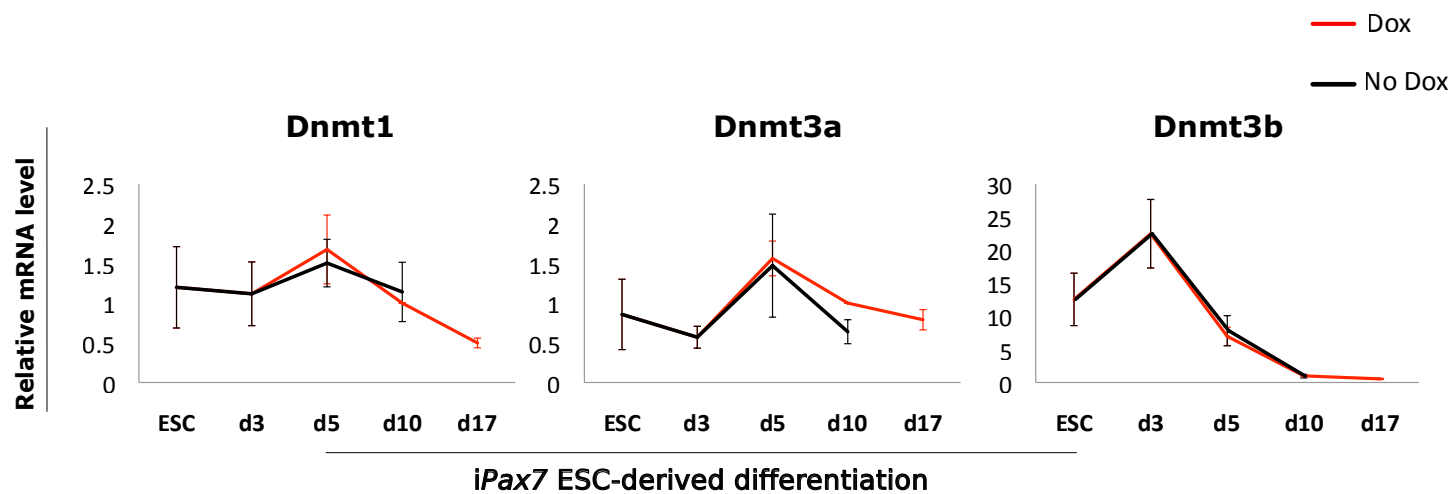**B**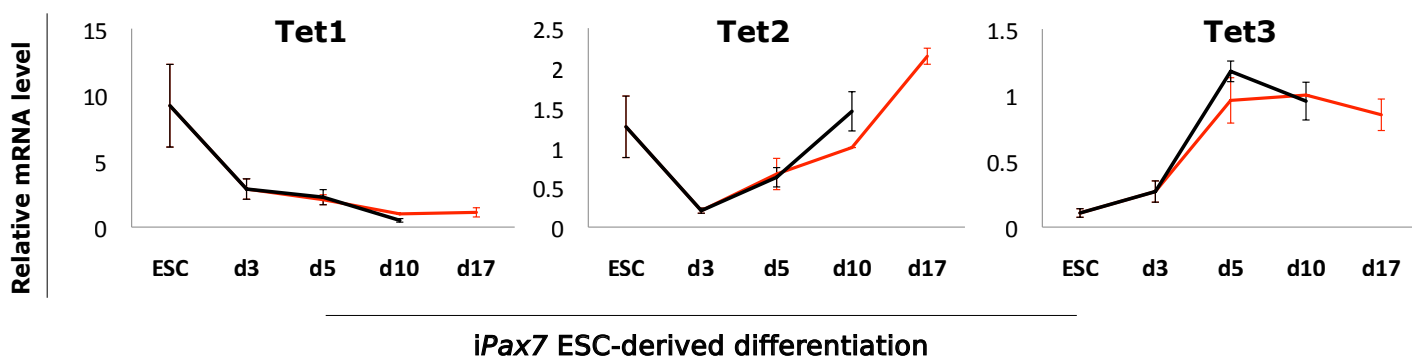**C**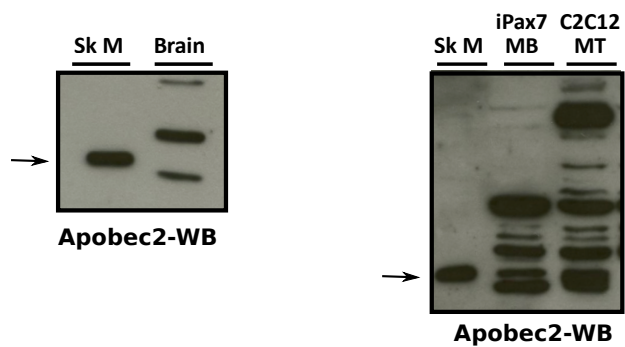

Supplement: Additional file 4: — Dnmt and Tet expression profiles during Pax7-induced ESC derived myogenic model and Apobec2 western blot analysis. Expression levels of Dnmt1, Dnmt3a and Dnmt3b (a) and Tet1, Tet2 and Tet3 (b) were measured by qRT-PCR at the successive time points of iPax7 ESC-derived myogenic model, with or without doxycycline, and normalized by Gapdh expression (n = 3, mean ± SD). c. Apobec2 western blots were performed on total protein extracts from skeletal muscle tissue (SK M) and brain (left), and muscle progenitor cells (iPax7-MB) and mature myotubes (C2C12-MT) (right). Skeletal muscle shows a specific Apobec2 band (32 kDa), whereas multiple bands are detected in brain tissue and in muscle cells. (PDF 798 kb) [file 12915_2016_250_MOESM4_ESM.pdf]

**A**

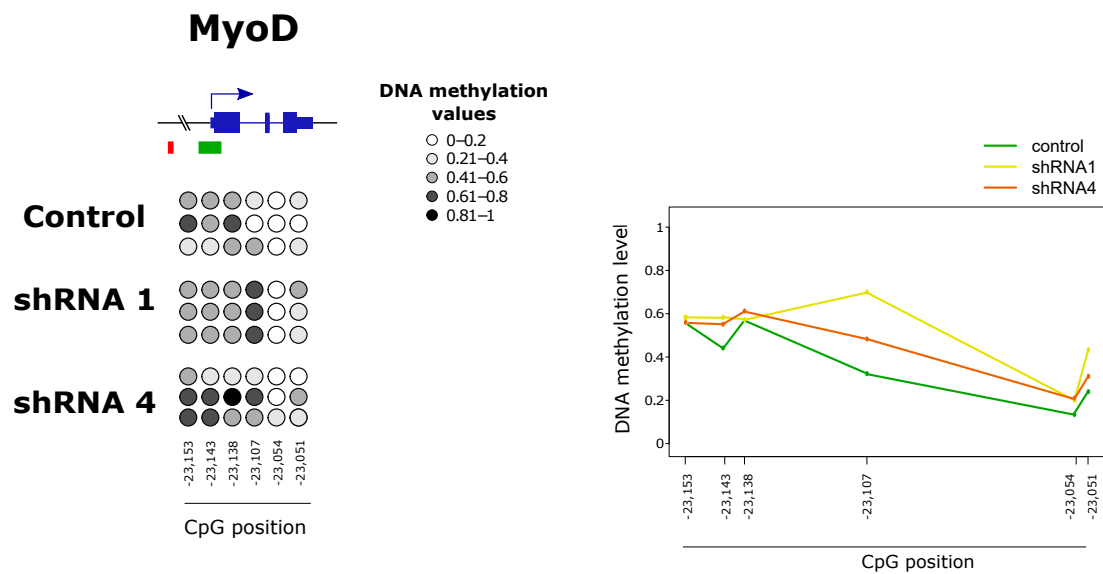

**B**

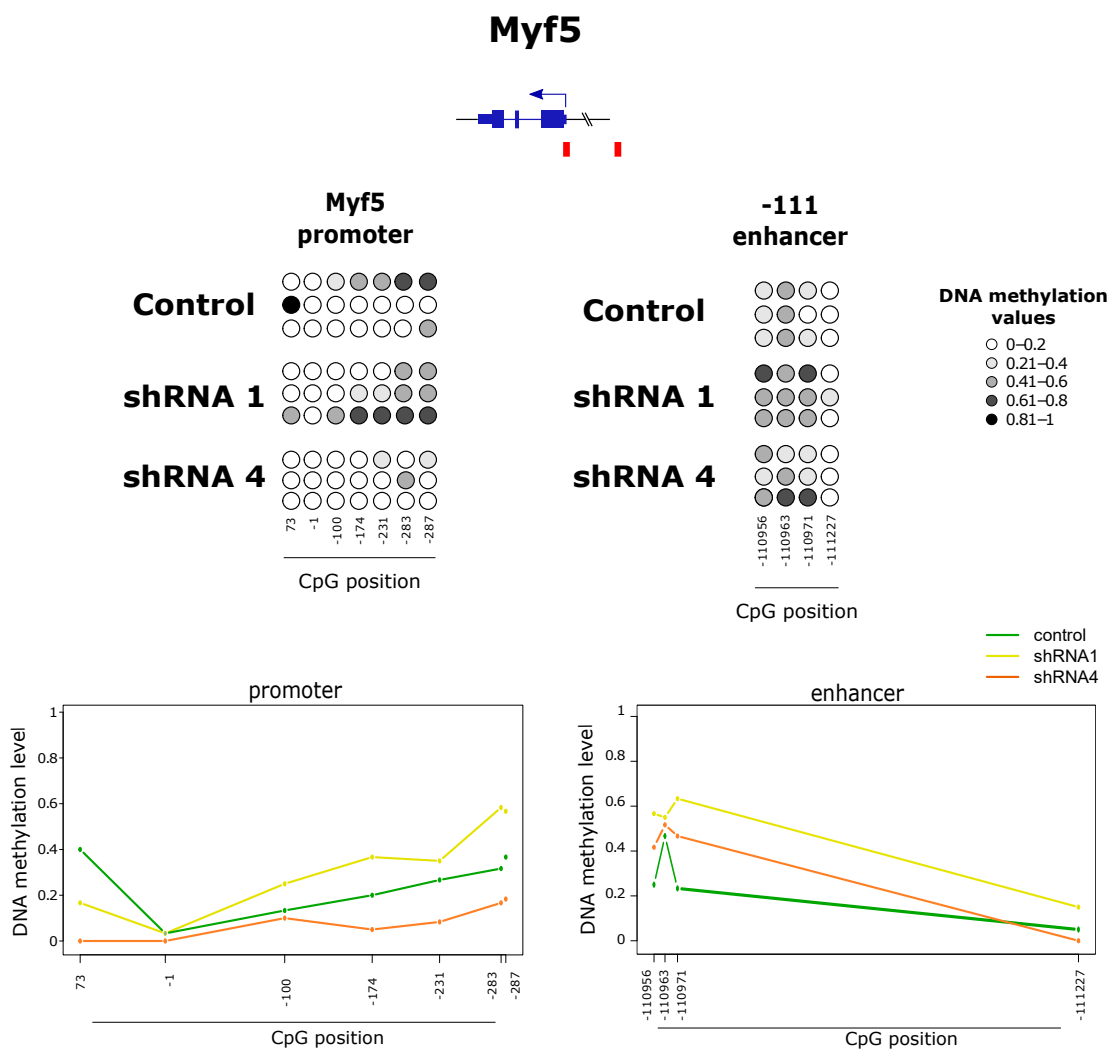

Supplement: Additional file 5: — DNA methylation analysis of MyoD and Myf5 genes in Apobec2 depleted cells. DNA methylation analysis by sodium bisulphite sequencing of the -20 kb MyoD enhancer (a) and promoter and -111 kb enhancer of Myf5 gene (b) in Pax7 ES-derived myogenic precursors transduced with Apobec2 shRNA1 and shRNA4, in three biological replicates. Each circle represents a CpG dinucleotide and its distance to the gene TSS is indicated below. The colour gradient represents the methylation level indicated in the legend. (PDF 100 kb) [file 12915_2016_250_MOESM5_ESM.pdf]
