# Supplementary material for: Muscle cell identity requires Pax7-mediated lineage-specific DNA demethylation
Source: BMC Biol. 2016 Apr 13;14:30. doi: 10.1186/s12915-016-0250-9 (PMC4831197; doi:10.1186/s12915-016-0250-9)
Supplement: Additional file 6: — Primer sequences used by qRT-PCR expression analysis. (DOCX 12 kb) [file 12915_2016_250_MOESM6_ESM.docx]

**Additional file 6**: qRT-PCR expression primer sequences 5' -> 3'

| **Gene** | **Forward** | **Reverse** |
| --- | --- | --- |
| 18S | TTGACGGAAGGGCACCACCAG | GCACCACCACCCACGGAATCG |
| Apobec2 | GACCCTGAGAAGCTGAAAGAG | CGACCACATAGCAGAGAAAGG |
| Ckm | AGGCATGGCCCGAGAC | AGATCACGCGAAGGTGGTC |
| Dnmt1 | CTCAGGGACCATATCTGCAAG | GGTGTACTGTAGCTTATGGGC |
| Dnmt3a | GGTCATGTGGTTCGGAGATG | AGGACTTCGTAGATGGCTTTG |
| Dnmt3b | GTACCCCATCAGTTGACTTGAG | TTGATCTTTCCCCACACGAG |
| Gadph | ACTCCCACTCTTCCACCTTC | TCTTGCTCAGTGTCCTTGC |
| Mrf4 | ATGGACCTTTTTGAAACTGGCTCC | CTGGCCAGGGCAGTGGGGAGGCTG |
| Myf5 | GCCATCCGCTACATTGAGAG | ACAGGGCTGTTACATTCAGG |
| Myh1 | CTCCAGGCTGCTTTAGAGGAA | CCTGCTCCTAATCTCAGCATCC |
| Myh4 | AAACCACCTCAGAGTTGTGGA | CTTCCGAAGGTTCCTGATTGC |
| Myh8 | AACAGAAACGCAATGCTGAGG | TCGCCTGTAATTTGTCCACCA |
| MyoD | GCCGCCTGAGCAAAGTGAATG | CAGCGGTCCAGGTGCGTAGAAG |
| Myogenin | GGTGTGTAAGAGGAAGTCTGTG | TAGGCGCTCAATGTACTGGAT |
| Pax3 | GAGCGAAGCTGCCCCCAG | GCCGTTGATAAATACTCCTCC |
| Pax7 | CAGGAGACTGCGTCCATCCG | CCGAACTTGATTCTGAGCAC |
| Tet1 | GACCGAAGATGTACCCTCAAC | CCTCCCAAACTTACAGCCG |
| Tet2 | AACCTGGCTACTGTCATTGCTCCA | ATGTTCTGCTGGTCTCTGTGGGAA |
| Tet3 | TCCGGATTGAGAAGGTCATC | TCCGGATTGAGAAGGTCATC |
