# Supplementary material for: Muscle cell identity requires Pax7-mediated lineage-specific DNA demethylation
Source: BMC Biol. 2016 Apr 13;14:30. doi: 10.1186/s12915-016-0250-9 (PMC4831197; doi:10.1186/s12915-016-0250-9)
Supplement: Additional file 7: — Primer sequences used by assessing the DNA methylation profiles of different genomic regions either by Sanger sequencing or pyrosequencing. (DOCX 16 kb) [file 12915_2016_250_MOESM7_ESM.docx]

**Additional file 7:** Sodium bisulphite sequencing primer sequences 5' -> 3'

|  | | |  |  |  |
| --- | --- | --- | --- | --- | --- |
| **Gene** | **Region** | **Forward (first PCR)** | **Reverse (first PCR)** | **Forward (nested PCR)** | **Reverse (nested PCR)** |
| Pax3 | +7587 +7454 | TTTAGATGAAAGTGAGAGG | TTCCTACTCCAACACCCTAA | GATATAAAGGAGGTAATTGAGT | ACACCCTAATAAAAAAAAAAATA |
| Pax3 | +644 +424 | AGGTGAATAGAAAGAGAAT | ACTACCCCCAAAATAAC | AGGTGAATAGAAAGAGAAT | ACTATACCCAAAATAATACAA |
| Pax3 | -96 -12 | TTAAAAAAGGTTAGAGAGGG | AACTCAAAACTCCTAATCAA | TAG TTG GGG TTA TTG GTA AA | TAAAAAAACTAATAAATACTCC |
| Pax3 | -728 -747 | TGGTATAGTTATGATAGAGA | AATCTTTACAACACTCCTAA | TAAGTTGTAAGTAATGGGGA | AACACTTCAACTCCTAACC |
| Pax3 | -5859 -5667 | TATAGTGGAATAAGTTTATATT | CTTACCTCTTCTCCTAA | GAGAAGTAGGGTAGAGTT | CCAAAAAAAAACCAAAAATCA |
| Pax3 | -6686 -6876 | GAAAGAATGAAGGTATAT | TCTCCCACAATAAAAACTA | GTATTGTTTAGAGAATATTTT | AATAAAAAAACCTTAAAAC |
| Pax3 | -18774 -19025 | GGGGTAATAGTTAGTTAGT | ATAACCTAAAAATATACAAAAAA | AGAAGATGAAAATGATTGTTTG | ATTCCTAAAACCTAAAACTTAAA |
| Pax3 | -20296 -202607 | GAGTTGTAGTTGGTTTAA | ATACCTAAATAAAAAAAATTAA | GTAGAAGGTGTGTAGGTA | AAATAAATAAACCAAAAAACC |
| Pax7 | +1400 + 1066 | TAAAAGTAGGTATTTGAAATTA | CCCCTCTTATCATTATAT | TAAAAGTAGGTATTTGAAATTA | CTAAAAAAAAAACCCCCTC |
| Pax7 | +931 +676 | ATATAATGATAAGAGGGG | TTCCACTCCACCAAAC | GTTTTTAATTGTTTTTGAGATAT | AATACATAATACCTTATTTCCc |
| Pax7 | +634 +456 | GGAAATAAGGTATTATGTAT | TCTACTAAATCCCAATCTC | GGAAATAAGGTATTATGTAT | CTCTTCTCTAACACAACA |
| Pax7 | +4588 +4391 | GAGGTATAGGATTGTGTTA | CAACTTATACTCTCCCTTT | GTAGTTTTATTTTTAGGATTTG | CCTTTTACCTTTCATTTCTAA |
| Pax7 | -7068 -1066 | TTTAAGTTTTTTGTAAGAG | ATTCTCTCCATACCCATTAA | GTAAGTTATTAAAGATAAAAATA | TTAAAACAAATCAAAAAATACC |
| MyoD | enhancer -20kb | ATTGAGAGTTAGGTAGGG | AACCAACTCACTTTCTCC | TTATAATATAGTTAGTTGGGG | CTAACCTCTCATACCTAATA |
| MyoD | enhancer -5kb 5' | TGGTGGTAGGTAGTTTTAGGT | TATTAATCTAACTAAACCTCTAA | AAAGTTTAGGGTTAGAGATTGAAT | ATATTACACCATAAAAAAACAAC |
| MyoD | enhancer -5kb 3' | TTGGAAGATTAGTTAAGGGAGTT | CAAAACAAAACAAACACACAAATA | GGGAGTTGAAATGTAAGGT | AAACACACAAATAACAAAAACC |
| Myf5 | enhancer | GTATGTTAGTATTATTTTT | CAAACTCACCTTTCAAC | TATAGTGTGTGATAAGTTAT | ATAACCCTAACCCACATAA |
| Myf5 | Promoter | TAGGAGTTTTTATAGAAGTATTT | AACCAACCCCAACCCCTT | AGTATTTAGAAGGGGAGAA | TTTATCCAAAAAACCACCA |
| Myf6 | Promoter | AGTAATGGTTATTGTTTATGA | TAAAACTCAATCCAACTTCTAA | TATTGGAAATATTAATGAGG | TAACTAATCTAATCTAAATAAT |
| Myog | Promoter | AAGAGAAGGTTAAGTGGA | AAACCCTAAAAATAAACAAAAAA | TTGGATTATGGAGGAGAGA | AATATCTCATACAACTCC |
| Ckm | Promoter | GGTTTTTTTAGTTGTTAATGT | ACCCCAATAATTTCTCTCAA | TTTGGGGGTTAGGGTTTA | TATCTATACTATAAAAATAATAA |
| Myh1 | Promoter | GGAGGAAGTAATAGTTGTT | ACACTCTTACCTTAAAACTT | GTTATATTGAGGAGTAGAAAG | TAAAAAAAAACAAATCACTCTC |
| Myh4 | Promoter | TTAGTTATAGTGTTAGATTTA | CACCCCAACTTCACTTTT | AATGGTTTTAAGTATTAGTAGA | CCTCAACTATCCTAACTAC |
| Myh8 | Promoter | TTTAAGGGAATGTAGTGTGG | ATCACTTACCTCTAAACTCTT | GAATAAATAAGGAAAGTGAGTT | AATAAAATATAAAAAACAACCCTA |
|  |  |  |  |  |  |
| Specific pyrosequencing primer sequences 5' -> 3' | | |  |  |  |
| **Gene** | **Region** | **Nested primer** | | **Sequencing primers** | |
| Myh1 | Promoter | TAAAAAAAAACAAATCACTCTC biotinilated reverse Primer for nested PCR | | ATTTTAGGTAATAAAGTG, TAGAATTTAGTAGTTTTTGAA | |
| Myh8 | Promoter | GAATAAATAAGGAAAGTGAGTT biotinilated forward Primer for nested PCR | | CCAAACACTTAACAACAAAA, TTATATTCAATTTAACAAAC | |
