# Supplementary material for: Muscle cell identity requires Pax7-mediated lineage-specific DNA demethylation
Source: BMC Biol. 2016 Apr 13;14:30. doi: 10.1186/s12915-016-0250-9 (PMC4831197; doi:10.1186/s12915-016-0250-9)
Supplement: Additional file 8: — Explanation and validation of the Sanger sequencing-based DNA methylation method. a. Quantification of DNA methylation state by bisulphite conversion followed by Sanger direct sequencing. DNA methylation state of each cytosine was analysed comparing the DNA reference sequence with the raw sequencing electropherograms of the bisulphite-converted sequence, visualized with Geospiza FinchTV software. The methylation level was ranked in five different intervals 0–0.2, 0.21–0.4, 0.41–0.6, 0.61–0.8 and 0.81–1, which reflects the DNA methylation state of this particular cytosine. Detailed explanations about how the methylation interval was assigned for each cytosine are in the Methods section. b. Correlation between Sanger and Pyrosequencing results. In the left panels are shown the DNA methylation state of three CpGs located in Myh1 and Myh8 promoter regions, in two embryonic stem cells ESC 1-2 and two primary myogenic precursor cells MPC 1-2. For each CpG it is shown the raw chromatogram obtained by Sanger sequencing with our corresponding assigned methylation interval and the methylation value obtained by pyrosequencing, which is inside the interval in most of the cases (right top scatter plots). On the right bottom scatter plots are shown the high linear correlation between Sanger sequencing and pyrosequencing results, with R2 = 0,98 for Myh1 and R2 = 0,91 for Myh8. (PDF 2930 kb) [file 12915_2016_250_MOESM8_ESM.pdf]

A

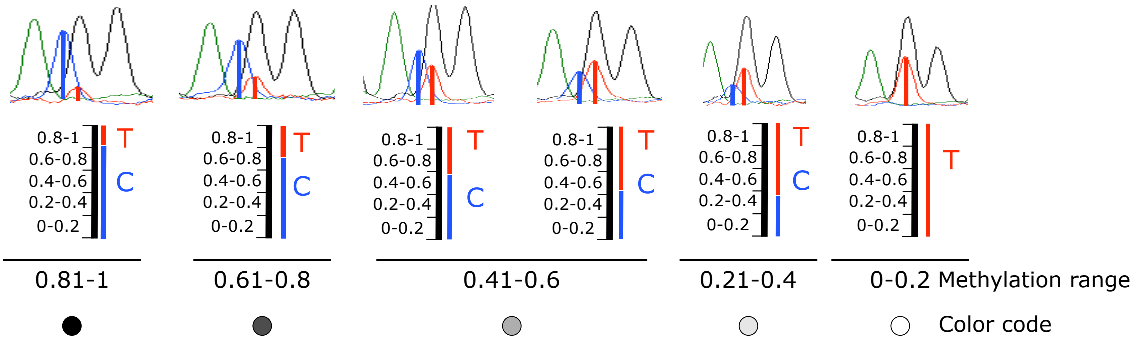

B

**Myh1**		**Analyzed CpG** Forward primer		
		**CpG # 1**	**CpG # 2**	**CpG # 3**
**Methylated sequence**		TACGG	GACGG	ATCGT
**Unmethylated sequence**		TATGG	GATGG	ATTGT
**Sample**	**Method**			
**ESC 1**	Sanger	0.6-0.8	0.8-1	0.6-0.8
	Pyro	0.53	0.68	0.62
**ESC 2**	Sanger	0.6-0.8	0.8-1	0.6-0.8
	Pyro	0.57	0.66	0.57
**MPC 1**	Sanger	0-0.2	0-0.2	0-0.2
	Pyro	0.04	0.04	0.00
**MPC 2**	Sanger	0-0.2	0-0.2	0-0.2
	Pyro	0.05	0.07	0.05

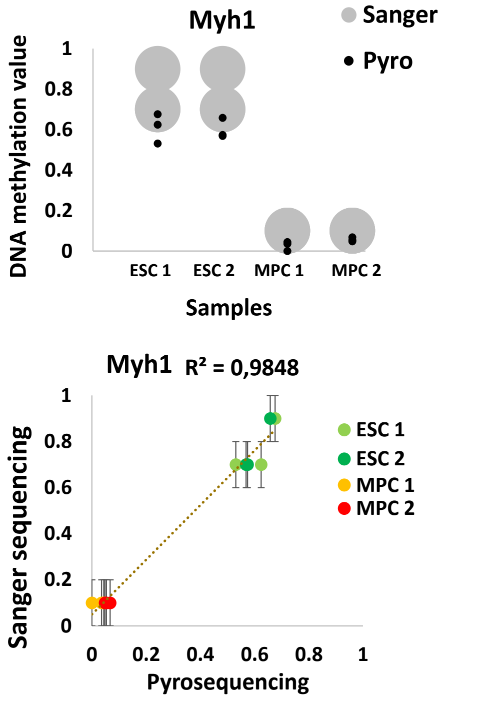

**Myh8**		**Analyzed CpG** Reverse primer		
		**CpG # 1**	**CpG # 2**	**CpG # 3**
**Methylated sequence**		ACGCC	TTCGAA	CTCGA
**Unmethylated sequence**		ACACC	TTCAAA	CTCAA
**Samples**	**Method**			
**ESC 1**	Sanger	0.8-1	0.8-1	0.8-1
	Pyro	0.75	0.78	0.83
**ESC 2**	Sanger	0.8-1	0.6-0.8	0.6-0.8
	Pyro	0.71	0.57	0.73
**MPC 1**	Sanger	0-0.2	0-0.2	0-0.2
	Pyro	0.23	0.16	0.19
**MPC 2**	Sanger	0-0.2	0.2-0.4	0-0.2
	Pyro	0.22	0.47	0.04

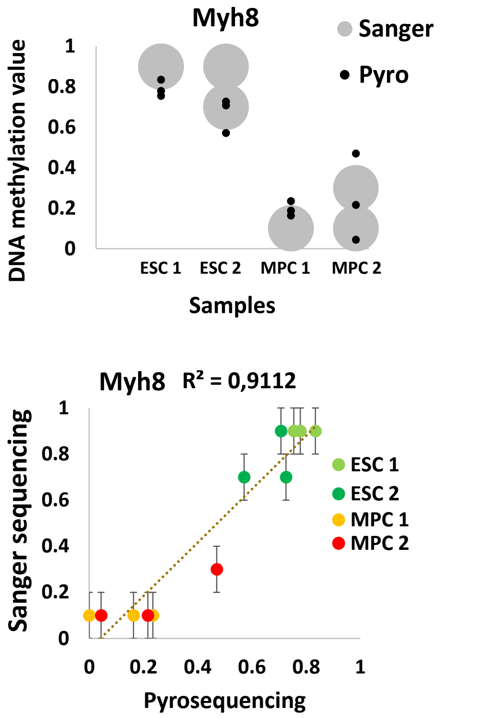

Supp. Figure 6
Carrió et al.
