# Supplementary material for: Muscle cell identity requires Pax7-mediated lineage-specific DNA demethylation
Source: BMC Biol. 2016 Apr 13;14:30. doi: 10.1186/s12915-016-0250-9 (PMC4831197; doi:10.1186/s12915-016-0250-9)
Supplement: Additional file 9: — GEO accession numbers for histone marks ChIP-seq data generated by the ENCODE Project Consortium and Dynlacht’s laboratory. (DOCX 43 kb) [file 12915_2016_250_MOESM9_ESM.docx]

**Additional file 9**: GEO accession numbers

| **Sample** | **ChIP-seq** | **GEO accession number** |
| --- | --- | --- |
| ESC | H3K4me1 | GSM769009 |
| ESC | H3K4me3 | GSM769008 |
| ESC | H3K27me3 | GSM1000089 |
| ESC | H3K27Ac | GSM1000099 |
| MB | H3K4me1 | GSM721288 |
| MB | H3K4me3 | GSM918415 |
| MB | H3K27me3 | GSM918408 |
| MB | H3K27Ac | GSE37525 |
| MT | H3K4me1 | GSM721289 |
| MT | H3K4me3 | GSM918416 |
| MT | H3K27me3 | GSM918414 |
| MT | H3K27Ac | GSE37525 |
